# Supplementary material for: Translation and validation of the Chinese version of EORTC QLQ-SWB32 assessing the spiritual wellbeing of women with gynecological cancer
Source: PLoS One. 2025 Apr 15;20(4):e0321790. doi: 10.1371/journal.pone.0321790 (PMC11999153; doi:10.1371/journal.pone.0321790)
Supplement: S2 Appendix — (PDF) [file pone.0321790.s002.pdf]

EORTC QLQ-SWB32

请您亲自回答所有的陈述，并在最符合您情况的数字上画圈。答案没有正确和错误之分。您所提供的信息将会被严格保密。

在过去一周里:	一点也不	有一点	很多	非常多
1. 我觉得自己有能力处理问题	1	2	3	4
2. 我觉得自己是平和的	1	2	3	4
3. 我能找到自己喜欢做的事情	1	2	3	4
4. 我觉得能原谅自己做过的事情	1	2	3	4
5. 我感到烦恼	1	2	3	4
6. 我感到孤单	1	2	3	4
7. 我担心对我来说重要的人的未来	1	2	3	4
8. 我觉得能与亲近的人分享关于生活的想法	1	2	3	4
9. 我能感受到对我来说重要的人的爱	1	2	3	4
10. 我觉得有人可以听我倾诉我的感受	1	2	3	4
11. 我感到能够信任他人	1	2	3	4
12. 我觉得能原谅他人做过的事情	1	2	3	4
13. 我觉得我作为一个人是被重视的	1	2	3	4
14. 我觉得我的人生是充实的	1	2	3	4
15. 我觉得我的人生是值得的	1	2	3	4
16. 我觉得能计划自己的未来	1	2	3	4
17. 我对未来有过担心和/或忧虑	1	2	3	4
18. 我在想是否还有什么事是能为我做的	1	2	3	4
19. 我觉得自己生病是不公平的	1	2	3	4
20. 我有时间静心、祈祷或者冥想	1	2	3	4
21. 我觉得别人为我祈祷是重要的	1	2	3	4

SPECIMEN

请接下页

这一页更关注您的总体想法与感受

总体而言:	一点也不	有一点	很多	非常多
22. 我相信神或比我更伟大的人或事	1	2	3	4
23. 我一直相信神或比我更伟大的人或事	1	2	3	4

如果第 22 和 23 题您的答案均为“一点也不”（第一个选项），请跳至第 27 题
否则，请连续作答

24. 我的信仰**在生病后**改变了	1	2	3	4
25. 我的信仰**在过去几周里**改变了	1	2	3	4
26. 我觉得与神或者比我更伟大的人或事之间有联结	1	2	3	4

总体而言:	一点也不	有一点	很多	非常多
27. 我觉得我会通过我的言语、行为和/或对他人的影响继续活下去	1	2	3	4
28. 我对生命的感受**在生病后**改变了	1	2	3	4
29. 我对生命的感受**在过去的几周里**改变了	1	2	3	4
30. 我相信人死后还有生命	1	2	3	4
31. 我的灵性健康状态良好	1	2	3	4

32. 您如何评价自己的整体灵性健康状态？ *请圈出下面的一个数字*

0	1	2	3	4	5	6	7
不知道/不能回答	**非常差**						**非常好**

感谢您完成这份问卷

如果您有其他的想法或评论，请写在下方

© Copyright 2014 EORTC Quality of Life Group. 版权所有 · 版本 6.3

SPECIMEN
