# Supplementary material for: Translation and validation of the Chinese version of EORTC QLQ-SWB32 assessing the spiritual wellbeing of women with gynecological cancer
Source: PLoS One. 2025 Apr 15;20(4):e0321790. doi: 10.1371/journal.pone.0321790 (PMC11999153; doi:10.1371/journal.pone.0321790)
Supplement: S3 Appendix — (PDF) [file pone.0321790.s003.pdf]

EORTC QLQ-SWB32

Please respond to all of the statements yourself by circling the number that best applies to you. There are no “right” or “wrong” answers. The information that you provide will remain strictly confidential.

During the past week:	Not at all	A little	Quite a bit	Very much
1. I have felt able to deal with problems	1	2	3	4
2. I have felt at peace with myself	1	2	3	4
3. I have been able to find things I enjoy doing	1	2	3	4
4. I have felt able to forgive myself for things I have done	1	2	3	4
5. I have felt troubled	1	2	3	4
6. I have felt lonely	1	2	3	4
7. I have worried about the future of people who are important to me	1	2	3	4
8. I have felt able to share thoughts about life with people who are close to me	1	2	3	4
9. I have felt loved by those who are important to me	1	2	3	4
10. I have felt that I have someone to talk to about my feelings	1	2	3	4
11. I have felt able to trust others	1	2	3	4
12. I have felt able to forgive others for things they have done	1	2	3	4
13. I have felt that I am valued as a person	1	2	3	4
14. I have felt that my life is fulfilling	1	2	3	4
15. I have felt that my life is worthwhile	1	2	3	4
16. I have felt able to plan for the future	1	2	3	4
17. I have had worries and/or concerns about the future	1	2	3	4
18. I have wondered whether anything can be done for me	1	2	3	4
19. I have felt that it is unfair that I am ill	1	2	3	4
20. I have had time for quietness, prayer or meditation	1	2	3	4
21. I have felt that it is important that other people pray for me	1	2	3	4

SPECIMEN

Please go to the next page

<u>This page focuses on your thoughts and feelings MORE GENERALLY</u>

**More generally:**	**Not at all**	**A little**	**Quite a bit**	**Very much**
22. I believe in God or in someone or something greater than myself	1	2	3	4
23. I have always believed in God or in someone or something greater than myself	1	2	3	4

If you answered BOTH items 22 AND 23 "Not at all" (option 1), please go to ITEM 27. Otherwise, please continue.

24. My beliefs have changed **since I have felt less well**	1	2	3	4
25. My beliefs have changed **in the last few weeks**	1	2	3	4
26. I feel connected to God or to someone or something greater than myself	1	2	3	4

**More generally:**	**Not at all**	**A little**	**Quite a bit**	**Very much**
27. I feel that I will live on through my words, deeds and/or influence on other people	1	2	3	4
28. My feelings about life have changed **since I have felt less well**	1	2	3	4
29. My feelings about life have changed **in the last few weeks**	1	2	3	4
30. I believe in life after death	1	2	3	4
31. I have spiritual wellbeing	1	2	3	4

32. How would you rate your overall spiritual wellbeing? *Please circle ONE number below.*

**0**	**1**	**2**	**3**	**4**	**5**	**6**	**7**
Don t know/ Can't answer	**Very poor**						**Excellent**

Thank you for completing this measure.

If you would like to make any further comments, please write them below.

SPECIMEN

© Copyright 2014 EORTC Quality of Life Group. All rights reserved. Version 6.3
